# A Standardized Rat Model to Study Peri-implantitis of Transmucosal Osseointegrated Implants

**DOI:** 10.34133/bmr.0021

**Published:** 2024-06-01

**Authors:** Xingchen Liu, Shudan Deng, Xiyan Li, Haiwen Liu, Zhixin Li, You Wu, Pu Luo, Xinyi Zhong, Ruoxuan Huang, Runheng Liu, Xiayi Wu, Baoxin Huang, Zetao Chen, Zhuofan Chen, Shoucheng Chen

**Affiliations:** ^1^ Hospital of Stomatology, Guanghua School of Stomatology, Sun Yat-sen University and Guangdong Provincial Key Laboratory of Stomatology and Guangdong Research Center for Dental and Cranial Rehabilitation and Material Engineering, Guangzhou, 510055, China.; ^2^ Department of Stomatology, The Eighth Affiliated Hospital, Sun Yat-sen University, Shenzhen, 518033, China.

## Abstract

With the high incidence rate, distinctive implant characteristic and unique infection pattern, peri-implantitis (PI) requires a specially designed implant animal model for the researches on the pathogenesis and treatments. Previous small-animal PI models exhibit variability in implant site selection, design, and surgical procedures resulting in unnecessary tissue damage and less effectivity. Herein, a quantitative-analysis-based standardized rat model for transmucosal PI-related research was proposed. After dissecting the anatomic structures of the rat maxilla, we determined that placing the implant anterior to the molars in the rat maxilla streamlined the experimental period and enhanced animal welfare. We standardized the model by controlling the rat strain, gender, and size. The customized implant and a series of matched surgical instruments were appropriately designed. A clear, step-by-step surgical process was established. These designs ensured the success rate, stability, and replicability of the model. Each validation method confirmed the successful construction of the model. This study proposed a quantitative-analysis-based standardized transmucosal PI rat model with improved animal welfare and reliable procedures. This model could provide efficient in vivo insights to study the pathogenesis and treatments of PI and preliminary screening data for further large-animal and clinical trials.

## Introduction

**Fig. 1.**
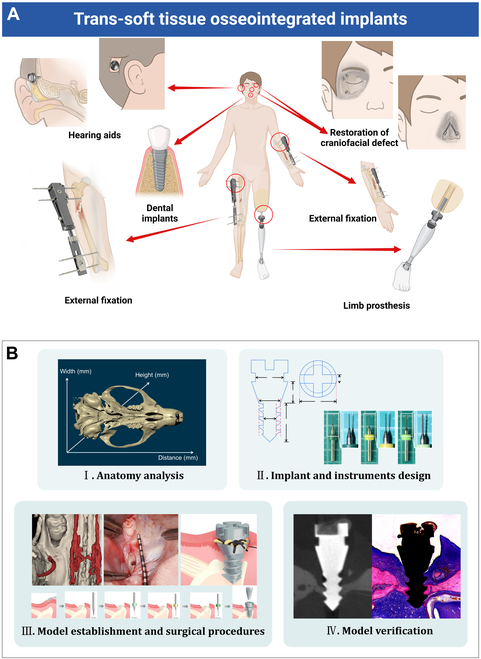
TOIs application schematic diagram and the research model construction flowchart. (A) TOIs application in the human body. (B) Establishment of standardized rat model for transmucosal osseointegrated implants PI research: I. Anatomy and available bone volume analysis of rat maxilla by micro-CT to select implant region. II. Implant and instruments design based on implant region anatomy. III. Model establishment and standard surgical procedures. IV. Model verification by micro-CT, qPCR, and histology.

Trans-soft tissue osseointegrated implants (TOIs), including orthopedic and oral implants, as well as hearing aids and craniofacial restoration implants, are increasingly used in the medical field (Fig. 1A). As one of the most representative TOIs, transmucosal dental implants possess the advantages of better restoration function, providing bone perception, and improving overall patient satisfaction [1–5]. Despite these advantages, peri-implantitis (PI) because of bacterial colonization remains an unsolved problem and can lead to devastating complications and high risk of failure [6,7]. It has been reported that PI have become the main cause of transmucosal dental implant failure, with an incidence as high as 14% to 43.9% in 5 to 10 years [8]. Given that approximately 12 million osseointegrated oral implants are implanted each year worldwide, PI has been recognized as one of the foremost clinical issues anticipated to be encountered in the coming decades [9].

In light of the unique features of implantation and infection patterns, it is essential to develop tailored animal models to acquire a foundational comprehension of in vivo pathophysiological mechanisms and treatment efficacy for transmucosal PI [10]. Nowadays, a number of studies have utilized standardized PI models in large animals [11–18]. Considering the advantages of lower cost, and the availability of a diverse range of molecular biology tools [19,20], the importance of small-animal models in advancing PI research is undeniable. Despite a few of PI models in the maxilla of small animals have been reported, however, these studies usually lack standard criteria such as age, weight, and gender of the animals, implant site, implant design and surgical procedures, etc., resulting in low efficacy and low comparability [21,22]. Besides, most of these implant models need to perform tooth extraction, inevitably increasing the complexity of surgery and extending the entire experimental period. Furthermore, these models resulted in varying degrees of damaging the roots, maxillary sinuses, adjacent muscles, and vascular system, all of which can cause unnecessary harm to animals. In consideration of animal welfare ethics and research efficiency, it is imperative to develop an efficient, streamlined, reliable, and standardized rat model for studying PI in oral implants. This approach aims to overcome existing challenges and propel PI research forward.

Here, we propose a standardized rat model of transmucosal osseointegrated implants PI with minimal tissue damage, a short experimental period, clarified surgical procedures, and improved animal welfare. The bone volume and gingival soft tissue in the region anterior to rat maxillary molar were systematically evaluated for ideal implant site selection and to provide a reference for customized implant design. Additionally, soft tissue vascular perfusion assessment was performed to guide the surgical incision to minimize bleeding and damage to the animal. In addition, a clear surgical process was established through standardized surgical tools and deliberately designed operating procedures. Finally, comprehensive assessments including the expression of the biomarkers, micro-computed tomography (micro-CT) and histological and histopathological images were conducted to confirm the successful inducement of PI (Fig. 1B). This model could be utilized to study the pathogenesis and treatments of PI and preliminary screening before further large-animal studies/clinical trials. Given the structure and pathophysiology similarity, we also discussed and summarized the potential applications of the proposed model for the active development of TOIs that are plagued by infection-related complications.

## Materials and Methods

### Animals

Eleven male Sprague-Dawley rats (450 to 500 g, 12 to 13 wk old) were purchased from Guangdong Medical Laboratory Animal Center (Guangzhou, China). Animals were housed and bred in specific-pathogen-free facility. All experiments were performed with prior approval from the Institutional Animal Care and Use Committee of Sun Yat-sen University (approval no. SYSU-IACUC-2022-001137).

### Anatomical structure

To clarify the anatomy of region anterior to maxillary molar and determine the ideal implant site, 5 rats were euthanized with 200-mg/kg sodium pentobarbital via intraperitoneal injection. To image and analyze the maxilla, the entire heads were removed, and the maxillas were scanned using micro-CT (Skyscan 1276, Bruker). The images were scanned at a resolution of 10 μm per pixel, voltage 70 kV, and current 114 μA. Three-dimensional images were obtained, and bone parameters were analyzed using Mimics software (Materialize, Belgium), CTvox (Burker micro-CT), and DataViewer (Bruker micro-CT).

**Fig. 2.**
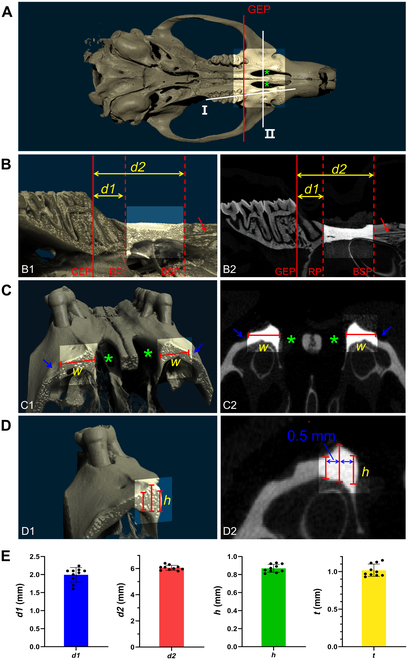
Anatomy analysis of the region anterior to the molar in rat maxilla. (A) Three-dimensional reconstruction model of rat maxilla. Line I is designed to obtain the sagittal view of the dentition and palatal area. Line II is designed to obtain the cross view of palatal area. GEP, gingival entry point. Green star, palatal cleft. (B) Sectional image of reconstruction model (B1) and micro-CT image (B2) of maxilla obtained from Line I section. *d1* means the subgingival extended distance of the mesial root of the maxillary first molar. *d2* means the distance between GEP and BSP. Red arrow, the bone suture. (C) Sectional image of reconstruction model (C1) and micro-CT image (C2) of maxilla obtained from Line II section. The blue arrow shows the buccal tendon attachment point, and the red line shows the available bone width (*w*). (D) Sectional image of reconstruction model (D1) and micro-CT image (D2) of maxilla obtained from Line II section. The red lines mean the available height of alveolar bone crest and the distance between the 2 adjacent red lines is 0.5 mm. (E) The measured value of *d1*, *d2*, *h*, and *t* (the thickness of soft tissue).

Several coronal planes were defined to measure the subgingival extended distance (*d1*) of the mesial root of the maxillary first molar. The most mesial point of tooth at the gingival margin level was defined as the gingival entry point, and the coronal plane cross the gingival entry point was called gingival entry plane (GEP) (Fig. 2A). The root plane (RP) was defined as a coronal plane tangent to the mesial root (Fig. 2B). So, the *d1* of mesial root can be calculated by measuring the distance between GEP and RP through CT.

To clarify the anterior boundary of implant region, the bone suture plane (BSP) was defined as a coronal plane tangent to the bone suture (Fig. 2B) and the distance between GEP and BSP called *d2*. Bone width and height were measured (Fig. 2C and D) within the region between RP and BSP.

### Implant site

The implant site was determined based on the anatomical position of the first molar mesial root and available bone volume of region anterior to maxillary first molar.

### Implant design

#### Soft tissue thickness measurement

In order to clarify the soft tissue thickness at the implant region, rats were euthanized with 200-mg/kg sodium pentobarbital via intraperitoneal injection, and the soft tissue was obtained from the implant site.

The thickness of the soft tissue at the ideal implant region was measured with a vernier caliper. The measurement standard is that the tissue can be clamped without falling and with no macroscopically visible extrusion of soft tissue (Fig. S1). Ten specimens were included and analyzed.

#### Implant design and fabrication

The implant size and shape were designed based on the bone volume and soft tissue thickness at the implant region chosen in advance. The implant was fabricated customized, measuring 3.8 mm in height and 1.7 mm in diameter. Ti-6Al-4V was used to fabricate implants (Shanghai Lingjian Precision Machinery Co., Ltd.), and a rough surface of the entire implant was produced by sandblasting (Al_2_O_3_, 110 μm, 280 kPa, 10 s) and acid etching (HCl, pH = 3, 20 min, 80 °C) [23].

### Model establishment

#### Vascular perfusion

The perfusate was prepared from normal saline, small-particle barium sulfate (YUNFU HONGZHI NEW MATERIALS CO., LTD, M-700), and gelatin (MAYA, China, 888891-714138) in a ratio of 20:5:1. The mixture was stirred at 37 °C for 2 h to form a white suspension. The perfusate were stored at 37 °C until further use.

The rats were deeply anesthetized through intraperitoneal administration of sodium pentobarbital. A thoracoabdominal incision was quickly made to expose the heart. The superior vena cava was cut to allow the perfusion fluid to exit. A 24G indwelling needle (Super Health Medical, China) was carefully inserted into the left ventricle. The vessels were sequentially flushed with heparinized saline (500 U/ml), 4% paraformaldehyde, and perfusate. All liquids injected into the vessel were preheated to 37 °C, and 50 ml of each liquid was injected per rat. Once the perfusion was completed, the superior vena cava was ligated and the rat bodies were stored at 4 °C overnight, and the intact heads of the rats were isolated to micro-CT scanning with the following parameters: source voltage 55 kV and current 145 μA. Images of hard tissue and vasculature of the rat heads were obtained. Three-dimensional images were acquired and analyzed using Mimics software (Materialize, Belgium).

#### Implant placement

Eleven male Sprague-Dawley rats weighing 450 to 500 g underwent surgery under general anesthesia. The rats were placed in a supine position, and the maxilla and mandible were kept separate using a mouth opener. A total of 22 implants with threaded tip were placed in the region anterior to bilateral maxillary molar. Postoperatively, penicillin (40,000 U/kg) and antondine (0.01 ml/kg) were applied and a soft diet was provided for 4 weeks to allow implant osseointegration.

#### Bacteria

*Porphyromonas gingivalis* ATCC 33277 (*P. gingivalis*) was cultured at 37 °C under anaerobic conditions. The bacteria in the logarithmic growth phase were used in the experiments at a concentration of 10^10^/ml and transported to the animal experiment site through anaerobic gas bag to maintain its anaerobic conditions and activity.

#### Inflammation induction

Four weeks after implant placement, osseointegration was evaluated clinically according to the presence/absence of the implant and the mobility when manually applying buccopalatal swing power to the implant, and the absence of implant mobility was considered as adequate osseointegration. Stable implants were randomly divided into inflammatory group (6 rats, 12 implants, *n* = 12) and control groups (5 rats, 9 implants, *n* = 9).

No inflammation induction was administered in the control group. Under general anesthesia, rats in the inflammatory group were underwent the inflammation induction surgery. 3-0 silk dipped with *P. gingivalis* solution (10^10^/ml) was fixed to the ligature groove of the implant. A normal diet was then administered postoperatively.

At the first day of the sixth, seventh, and eighth weeks after ligature, 500 μl of *P. gingivalis* solution (10^10^/ml) per implant was smeared over the silk respectively [16,24–28].

### Model verification

#### Micro-CT scanning

At the end of the eighth week after ligature, all rats were sacrificed, and the maxilla were removed for micro-CT scanning (source voltage 70 kV and current 114 μA) to assess the bone loss level around implants. Mimics software (Materialize, Belgium) was used for analysis. The degree of the bone defect was assessed in buccopalatal and mesiodistal direction by bone level measurement. The distance between the implant head (Fig. S2, line a) and the most coronal position of the marginal bone attached to the implant was measured and scaled to the actual length (Fig. S2). All measurements were performed in a double-blind manner by 2 independent researchers.

#### Quantitative reverse transcription polymerase chain reaction (RT-qPCR)

After sacrificing rats, a 2-mm width of peri-implant soft tissues were rapidly removed from maxillas and stored at −80 °C for further experimentation. The total RNA was extracted using RNAzol RT (Sigma-Aldrich, St. Louis, USA). The extracted total RNA was measured by Nanodrop (Thermo Fisher, USA) and then converted to complementary DNA by Hieff III 1^st^ strand cDNA Synthesis Kit (Yeasen, China). Aliquots of cDNA samples were loaded for reverse transcription polymerase chain reaction (RT-qPCR) analysis on an ABI 2-step system (Applied Biosystems, USA) using Hieff qPCR SYBR Green Master Mix (Yeasen, China). The expression level of interleukin-1β (IL-1β), IL-6, IL-18, and tumor necrosis factor-α (TNF-α) were detected. Primer sequences were shown in supplementary materials (Table S1). The gene expression was normalized to the relative abundance of the reference gene, glyceraldehyde 3-phosphate dehydrogenase, utilizing the 2^−ΔΔCt^ method.

#### Histological analysis

For further histological analysis, maxillary specimens were collected after rats were sacrificed and immersed in 4% paraformaldehyde for 48 h.

Four calcified maxillae specimens (2 specimens in control group, 2 specimens in inflammatory group) were dehydrated and embedded in methyl methacrylate (SHANGHAI ZHANYUN CHEMICAL Co., LTD.). Longitudinal histological sections (parallel to the long axis of the implant) were obtained by cutting specimens into 1 mm (Leica, SP1600) and grinding to a final thickness of 100 μm (LaiZhou Weiyi, MP-2B). Sections were stained with methylene blue and acid fuchsin and observed by a stereomicroscope (Leica, MZ10F).

The other specimens were decalcified in 15% EDTA for 4 weeks, and the implants were carefully unscrewed from the maxillas. Specimens were embedded and block-mounted in paraffin and cut into 4-μm sections along the sagittal or coronal plane for hematoxylin and eosin (H&E) and tartrate-resistant hydrochloric acid phosphatase (TRAP) staining.

All histological images were digitally captured (Aperio AT2, Leica, Germany) and analyzed using an Aperio Image Scope 12.3 (Leica Biosystems, Germany).

A region of interest (ROI) was selected from the sections and defined by a rectangle with the longer side parallel to the long axis of the implant (Fig. S3). The length of longer sides was equal to the overall length of the threaded section and transgingival segment of implant (i.e., 2.8 mm), and the longer sides of the rectangle were 500 μm away from the surface of implant. The specimens with other structures such as roots located within 500 μm around implants were not included in the statistics. Bone area (BA, *n* = 5 specimens/group) was measured within the ROI (Image pro plus 6.0).

To assess osteoclasts (OCs) numbers (*n* = 5 specimens/group, 1 histological section per specimen), OCs were counted along the implant and alveolar ridge within the ROI mentioned above. TRAP-positive multinuclear cells in contact with bone were considered OCs. The number of OCs per specimen was counted and averaged for each group to compare differences between the inflammatory and control groups.

To quantitatively compare the level of inflammatory cells infiltration, inflammatory cells in 4 squares (50 × 50 μm) of soft tissue above the bone surface were counted at 400× magnification (Fig. S3).

### Statistical analysis

All statistical analyses were conducted using GraphPad Prism software (Version 8.0.2). Differences between groups were assessed utilizing either 1-way or 2-way analysis of variance (ANOVA), followed by Tukey’s multiple comparisons post hoc test or least significant difference test. Data are presented as the mean ± standard deviation (SD). A significance level of 0.05 (α = 0.05) was predetermined. The significance levels between groups are denoted as follows: * (*P* < 0.05), ** (*P* < 0.01), and *** (*P* < 0.001), indicating progressively higher levels of significance.

## Results

### Anatomic study of region anterior to maxillary molar to determine ideal implant site

To investigate the detailed anatomy of region anterior to maxillary molar so that to determine the ideal implant site, a large quality of data was acquired to illustrate the hard and soft tissue structure.

The available bone dimensions (length, width, and height) of the region anterior to maxillary molar are constrained by anatomical landmarks, as depicted in Fig. 2A. To protect the mesial root of maxillary first molar, the distance (*d1*) between GEP and RP and the distance (*d2*) between GEP and BSP were measured (Fig. 2B). The distance (*d2*-*d1*) is set as the length of the implant region (Fig. 2B). The width of the implant region is determined by the distance from the buccal tendon attachment to the palatal cleft on the palatal side (Fig. 2C). There is a relatively more bone height at the alveolar bone crest. To ensure a greater height of bone around the implant, the implant site should be located on the alveolar bone crest as far as possible. We measured the bone height within a 0.5-mm range from the alveolar bone crest in the buccal-palatal aspect. The height is defined as the distance from the alveolar bone crest to the bone surface of the sinus cavity above (Fig. 2D).

According to the measurement by micro-CT, *d1* is 1.992 ± 0.202 mm, *d2* is 6.054 ± 0.191 mm, and *h* is 0.869 ± 0.044 mm (Fig. 2E). In summary, the ideal implant region was identified at a position 2 mm anterior to the maxillary first molar. Within this specific region, the available bone volumes were measured as follows: length ~ 4 mm, width ~ 1 mm, and height ~ 0.85 mm.

**Fig. 3.**
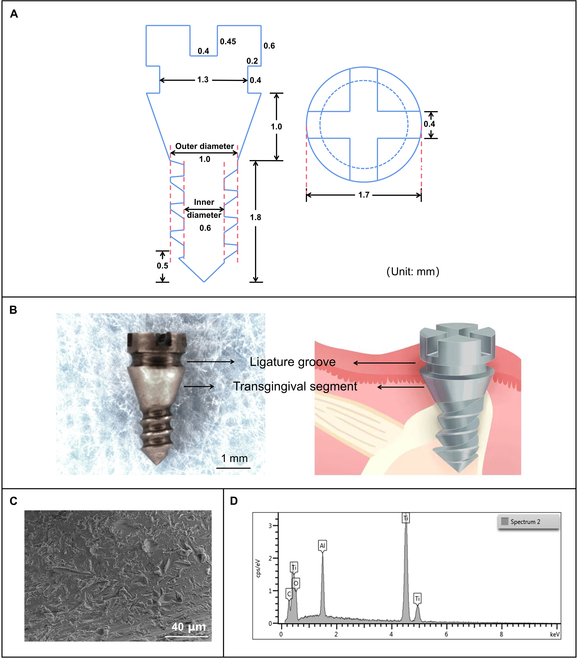
Structure and external design diagram of the implant and surface characterization. (A) Shape and dimension of implant. (B) Physical diagram and schematic diagram of implant. (C) Rough implant surface can be seen under a scanning electron microscope (SEM). (D) Energy-dispersive x-ray analysis of implant surface. The elemental composition of the implant surface is mainly Ti, O, Al, and C.

After selecting the ideal implant region, we measured the thickness of the soft tissue in this area. The averaged thickness of soft tissue is 1.017 ± 0.083 mm (Fig. 2E), which can guide the design of the transgingival segment of implant (Fig. 3A).

### Design of implants and operation instruments based on anatomic study

Based on the available bone volume of the implant site, the specific implant was designed (Fig. 3A). The total length of the threaded segment was 1.8 mm. The sharp tip increased the self-tapping for simple manual screw-in, which was 0.5 mm. The inner diameter of 0.6 mm in the thread segment ensures its strength, and the outer diameter of 1 mm meets the limit of bone volume. The thread depth of 0.2 mm ensures the initial stability of the implant.

Based on the thickness of the soft tissue at the implant site, a 1-mm height transgingival segment was designed to ensure the implant would not be covered by soft tissue (Fig. 3A and B). In addition, the expanded neck also could be used as a stop point for implantation to avoid insufficient or too much implant depth.

The ligature groove is convenient for ligature operation and makes the silk less likely to fall off (Fig. 3B). It avoids downward displacement of the silk, which ensures that the mechanism of inflammation is colonization of bacteria rather than foreign body mechanical damage, so that the model can better mimic the clinical situation. Sandblasting and acid etching of the implant resulted in a rough surface (Fig. 3C). The elemental composition of the implant surface after sandblasting and acid etching was mainly Ti, O, Al, and C (Fig. 3D and Fig. S4).

To achieve a precise operation to complete the surgery as designed and to be closer to the actual clinical situation, we designed a complete set of surgical tools, including various instruments and drugs (Fig. S5 and Table S2). In the assistance system, atropine can inhibit salivary secretion and adrenaline is used for local hemostasis. They play an important role in maintaining a clear surgical field, reducing bleeding and preventing accidental aspiration and choking cough to reduce the harm to animals. The special operating table enable efficient fixing of the rats, and the matching operating lamp help to provide a clearer visual field. The remaining devices are also used to clearly and safely expose the surgical field. The locating, implant site preparing, and cooling systems were highly similar to the tools used in the clinical practice. Owing to the lack of automatic screw-in equipment that matches with and can carry our implant, we performed manual screw-in with a customized screwdriver and use micro forceps to assist fixation (Fig. S5).

### Model establishment and surgical procedures

Before surgery, to minimize bleeding and reduce tissue damage, vascular perfusion was conducted to visualize the local blood vessel distribution within the soft tissues. A relatively great vessel is present in the soft tissues of the buccal side of region anterior to maxillary molar, extending from maxillary bone surface toward the mandible (Fig. S6). The position where the vessel reaches the surface of maxilla is approximately 2 mm mesial to the gingival entry point of the first molar and slightly buccal to the alveolar crest. On the palatal side of the alveolar ridge, great vessels are distributed at the surface of palatal cleft (Fig. S6). In addition, there is no large vessel distributes in the soft tissue above the alveolar ridge.

**Fig. 4.**
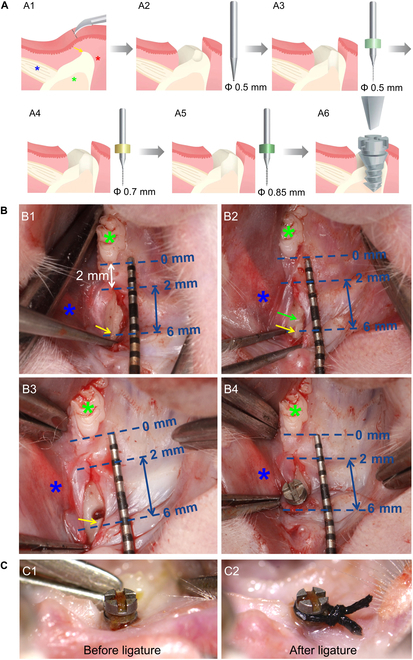
Surgical procedures flowchart for model construction and important anatomical landmarks. (A) Schematic diagram of surgical procedures, including surgical incision (A1), ball drill positioning (A2), implant hole sequential preparation (A3 to A5), and implant placement (A6). (B) Photographic image of surgical procedures, including surgical incision (B1), ball drill positioning (B2), implant hole preparation (B3), and implant placement (B4). Green star, the maxillary first molar; blue star, the buccal tendon; yellow arrow, the alveolar ridge. (C) Implant ligated by silk for inflammation induction.

The position of incision and implant region was shown in Fig. S7. To ensure the safety of animals and precision of the implant site, we designed the following surgical procedures including surgical incision, ball drill positioning, implant hole sequential preparation, and implant placement (Fig. 4A).

In detail, to avoid great vessels, surgical incision was set at 2 mm mesial to the first molar and slightly palatal to the extension line of the dentition (Fig. 4B1). The mucoperiosteal flaps were gently pulled apart to expose the alveolar bone crest, buccal tendon (Fig. 4B1). The 0.5-mm ball drill was used to position the implant site on the alveolar bone crest between 2 and 6 mm mesial to the first molar (Fig. 4B2). Generally, the tendon is visible on the buccal side in this region, which can be used as a reference for the mesial-distal position of the implant (Fig. 4B2). Then, the implant hole was sequentially prepared by twist drills in diameters of 0.5, 0.7, and 0.85 mm with rotation speed under 1,500 rpm and precooled normal saline (Fig. 4B3). The implants were manually screwed in using a screwdriver (Fig. 4B4). The bone height and soft tissue height after immediate implant placement were shown in Figs. S8 and S9.

Four weeks with soft food after implant placement, the peri-implant soft tissue has healed well without redness, swelling, and bleeding (Fig. 4C1). The ligature groove was exposed and ligated by the silk contaminated with *P. gingivalis*. In addition, silk knots were placed at the buccal side of implants (Fig. 4C2). The entire model establishment process was shown in Fig. S10.

### Model verification

To evaluate the success of this model, we performed clinical observations, micro-CT, RT-qPCR, and histological analysis.

Four weeks after implant surgery, all rats survived, 21 of the 22 implants were in place, and the implant survival rate was 95.45%. We then grouped them, with 6 rats and a total of 12 implants as the inflammatory group, 5 rats with 9 implants were used as the control group. Within 5 weeks after ligature, no rats died, and 1 implant in the inflammatory group was loosened and shed due to inflammatory bone resorption. During the procedure of applying bacteria on the first day of sixth, seventh, and eighth weeks after ligature, 1 rat died because of an anesthetic accident, resulting in the loss of 2 implants. Only 1 other implant of inflammatory group fell out naturally during these 3 weeks due to inflammatory bone resorption. At the conclusion of the whole experiment, 10 rats survived and 17 implants in place. The implant survival rate was 77.27% and was 85% if anesthesia accident not counted (Table).

**Table.** Implant survival

Time point	Event	Rats survival	Implants remaining (Stable; Mobile)		Number of death	Implants lost: Rats death; Other reasons
0 wk	Implants placement	11	22 (21; 1)		0	/
4 wk	Ligature	11	21 (21; 0)		0	1: 0; 1
Experimental grouping: 11 rats, 21 implants				Control group (CG): 5 rats, 9 implants		
				Inflammatory group (IG): 6 rats, 12 implants		
9 wk	The first time of *P.g* application	11	20 (20; 0)		0	1: 0, 1(IG)
12 wk	Sacrifice	10	17 (17; 0)		1	3: 2(IG), 1(IG)
Control group (CG): 5 rats, 9 implants						
Inflammatory group (IG): 5 rats, 8 implants						
		End of implant osseointegration (4 wk)			End of inflammatory procedure (12 wk)	
Implant survival rate		95.45%			77.27%	
Implant survival rate (without anesthesia accident)		95.45%			85%	

**Fig. 5.**
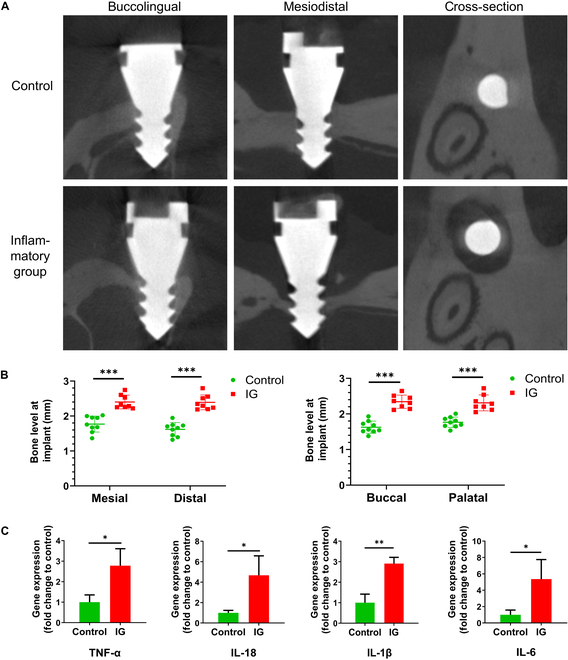
Results of inflammation model construction and expression of inflammatory factors. (A) Bone level in micro-CT. Compared to the control group, markedly lower bone levels were observed around implants in the inflammatory group in both buccopalatal direction and mesiodistal direction. (B) Comparison of bone level measurements in the control and inflammatory groups (***: *P* < 0.0001, 1-way ANOVA and least significant difference test). IG, inflammatory group. (C) IL-1β, IL-6, IL-18, and TNF-α expression levels are presented as fold changes compared to the control group. Data are shown as the mean ± SD (*P* < 0.05, *n* = 3, ANOVA and Tukey’s test).

The micro-CT results demonstrate that the inflammatory group exhibits markedly higher bone resorption in the mesial, distal, buccal, and palatal directions compared to the control group (*P* < 0.001, Fig. 5A and B). IL-1β, IL-6, IL-18, and TNF-α are indicative factors of inflammation associated with bone implants. The qPCR results show that the expression of these 4 cytokine genes were markedly higher in the inflammatory group compared to the control group (*P* < 0.05, Fig. 5C).

**Fig. 6.**
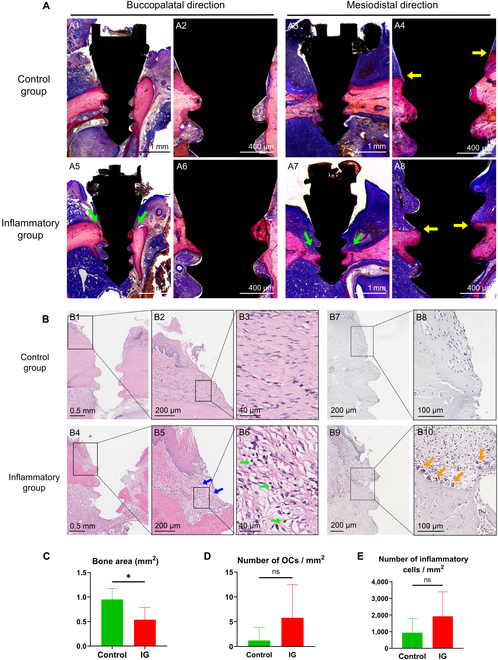
Histology analysis of the inflammation model. (A) Histological image of methylene blue and acid fuchsin staining of control group (A1 to A4) and inflammatory group (A5 to A8) after 8 weeks of implant placement. A1, A2, A5, A6 and A3, A4, A7, A8 are the images of buccopalatal sections and mesiodistal sections, respectively. Green arrow shows the pits form by bone absorption. Peri-implant bone level markedly reduced (yellow arrow) in inflammatory group. (B) Representative histological image of H&E staining (B1 to B6) and TRAP staining (B7 to B10) from mesiodistal direction. In the control group, the morphology of bone surface was regular and the soft tissue of bone surface is dense and with a little or without inflammatory cells infiltration. In the inflammatory group, bone surface was irregular with lots of pit and the soft tissue is edema (blue arrow) evidently with little or a large number of inflammatory cells (green arrow) infiltration. The OCs in inflammatory group is markedly more than control group (orange arrow). (C) The BA of histological image of control group is markedly larger than that of inflammatory group (*P* < 0.05). (D) The OCs in the inflammatory group are more than those in the control group but have no statistical significance (*P* = 0.1893). (E) The inflammatory cells infiltration in the inflammatory group is more than that in control group but has no statistical significance (*P* = 0.2323).

Hard tissue sections revealed that the inflammatory group exhibits bone resorption in the buccopalatal and mesiodistal directions (Fig. 6A). The absorbed areas of bone tissue have been replaced by soft tissue. Representative histological images of H&E staining from mesiodistal direction and buccopalatal direction are respectively shown in Fig. 6B and Fig. S11. In the inflammatory group, there is significant bone height reduction around the implants (Fig. 6B4), displaying concave bone resorption (Fig. 6B5). Partial replacement of bone by soft tissue were evident, accompanied by noticeable soft tissue swelling and infiltration of inflammatory cells (Fig. 6B6). TRAP staining showed more OCs on the alveolar bone surface in the inflammatory group (Fig. 6B7 to B10).

Peri-implant BA in ROI (Fig. S3) of control group is markedly larger than that of inflammatory group (*P* < 0.05, Fig. 6C) and the number of OCs in ROI of control group is markedly smaller than that of inflammatory group (*P* < 0.01, Fig. 6D). Compared with control group, the number of inflammatory cells in inflammatory group is markedly higher (*P* < 0.001, Fig. 6E).

## Discussion

TOIs are rapidly developed in the field of biomedical research and are widely used in osseointegrated prostheses of amputees, oral implants, bone anchor hearing aids, and repair of craniomaxillofacial defects [3–5,29,30]. Being a representative form of TOI, transmucosal osseointegrated dental implants offer superior restoration functionality, contribute to bone perception, and enhance overall patient satisfaction [1–5]. However, one of the most prevalent and severe complications of transmucosal osseointegrated dental implant is peri-implant infection [5], which leads to peri-implant bone resorption, loosens and detaches the implant, and causes failure. Therefore, it is particularly important to employ appropriate animal models for in vivo PI studies. The greatest advantage of the present study is that it provides a standard, convenient, and scientific animal model in which we clarified the anatomy of the rat maxillary premolar region, designed suitable implants, and developed a refined implant surgical approach to obtain a higher success rate of osseointegration and inflammation induction, as well as better animal welfare for transmucosal osseointegrated PI studies.

### Why was the region anterior to maxillary molar chosen?

#### Consideration of period of experiment

In the majority of studies, the maxillary molar region was chosen as the implant site [25,26,31]. However, this approach requires additional tooth extraction surgery, which increases the risk of death from anesthesia and prolongs the experimental period of this animal model by 8 weeks. In our model, implants were placed in a region anterior to maxillary molar, reducing tooth extraction procedures and shortening experimental periods and related surgical risk.

#### Consideration of local anatomic structures and surgical access

As the rat maxilla has a complex anatomy and the models of transmucosal osseointegrated implants include complex procedures such as implant placement and induction of inflammation, the implant site must be carefully selected to avoid damage to important anatomical structures, including sinus cavities, muscles, and nerves*.*

In a study by Yue et al. [32], though the implant site is also in a region anterior to maxillary molar, the incision was set vaguely and implants were placed randomly without clear descriptions and corresponding anatomical landmarks as a reference, resulting in some of implants placed on the roots of the first molar. In this model, a 2-mm mesial root space was reserved by fully understanding the anatomical structure near the implant site through micro-CT before surgery, which ensure that the adjacent teeth were not injured during implantation, thereby reducing damage to the animal and ensuring the osseointegration effect.

In the study by Becker et al. [33], the implant site was set in the palatal plate region of the maxilla. However, during our observation of soft tissue vascular perfusion, we found the presence of larger blood vessels within this area’s soft tissue. Consequently, it was inevitable that these vessels would be damaged during the surgical procedure, leading to significant animal injury. Moreover, the posterior positioning of the palatal plate region in the maxilla added to the complexity of the operation.

### How does one keep the model standardized?

To improve the success rate, stability, and comparability of research results, it is necessary to standardize the construction of the model. Four major factors are involved in determining the standardization: animals, implant and instruments, surgical procedures, and operators.

#### Animals

Rats of different genders, weights, and ages can exhibit variations in bone volume. Generally, bone volume tends to increase with age and weight, with males often demonstrating larger bone volumes compared to females, although this correlation is not strictly consistent [34,35]. In the study, to ensure the reference ability and reproducibility of the measured bone volume, the criteria were set to include male rats aged 12 weeks, with a body weight of 450 to 500 g, as the experimental subjects. Besides, if female rats are used, the secretion of estrogen may become another confounding factor affecting experimental result, thus decreasing the accuracy of the model in evaluating various PI-related research [36]. Therefore, in order to remove the unwanted influence of estrogen, male rats were preferentially considered for developing the standardized model.

In this study, we constructed the standardized model using the most commonly used Sprague-Dawley rats. However, whether our model is suitable for other species of rats, such as Zucker Fatty rat, to meet different research needs still needs further verification.

#### Implant and instruments

To keep the model standardized, both bone and soft tissue volumes should be considered to design a compatible implant and to obtain better osseointegration and suitable soft tissue penetration.

We designed implants of appropriate size according to the bone volume and soft tissue thickness at the selected implant site and combined them with maneuverability. Compared with 4-mm length implants [23,32,33], our implants cause less damage to the surrounding tissues and are easier to operate than 0.5-mm (diameter) × 1-mm (length) implants [24–27,31]. The sharp tip of the implant makes it easy to screw. The design of transgingival segment and ligature groove has been neglected in previous studies and has rarely been reported. Transgingival segment ensures that the implant can be exposed to the oral cavity and helps avoid implanting too deep. The ligature groove is designed so that the ligature cound not move up and down avoiding the controversy that the inflammation is due to foreign body irritation (Fig. 3B). The coronalapical position of the ligature and how frequently the ligature is replaced during plaque formation determine the rate and amount of bone resorption [28]. Therefore, uncertain coronal-apical position, uncertain detachment of the ligature, and religature time are considered as adverse factors for producing exact inflammation. In this study, the silk ligated in the ligature groove were all in place; therefore, the uncertainty mentioned previously was avoided and the fixed position of the ligature and the continuity of inflammation induction were guaranteed.

Besides, to achieve a precise operation to complete surgery as designed and to be closer to the clinical situation, we described a complete set of surgical tools, including various instruments and drugs (Fig. S5 and Table S2). In summary, these tools provide significant assistance to the procedures, ensuring a smooth surgical process.

#### The surgical procedures

A variety of factors, including surgical incision, ball-shaped drill positioning, implant hole sequential preparation, implant placement, and inflammation-inducing methods need to be carefully designed to enhance the efficiency and reliability of the model.

Compared with other similar studies with vague surgical procedures [20,24–28,31–33,37–39], we clearly defined detailed and reasonable surgical procedures. The incision was designed to cause less damage to the animal, minimize bleeding, and clearly expose the surgical region. Tools in the assistance system also helped to better expose the surgical field and facilitate the operation. The use of a periodontal probe and a small ball-shaped dental drill in the locating system helped precisely mark the implant site. The step-by-step implant hole preparation process further improve the accuracy of the implant site and axial correctness. Limiting rotate speed can reduce heat generation and irrigating cooling water can dissipate heat from drilling to avoid thermal osteonecrosis [40,41]. The limited rotation speed and irrigation of the precooled saline ensured cell viability and osseointegration. The difference in diameter between the final drill and the implant helped to the primary stability of the implant [42,43]. Small customized screwdrivers helped screw in the small implants. Feeding on a soft diet for 4 weeks after implant placement may help improve the survival rate of implants [28].

Ligature has been the common method of inducing PI to investigate the pathogenesis and therapy [16,31,37]. *P. gingivalis* is one of the major pathogens associated with PI and has been reported to have a reliable inflammatory induction effect [44,45]. This model ensured the success rate of inflammation induction by combining 2 effective methods. The design of the ligature groove in the neck of the implant also excluded inflammation caused by foreign body irritation, as mentioned previously. Some studies utilize carboxymethyl cellulose as a vehicle for the *P. gingivalis* solution to increase the viscosity so that the *P. gingivalis* can adhere to the surface better [46].

#### The operators

Different operators may have varying levels of experimental skills and experience, and there may be differences in the operational specifications and experimental procedures followed during the experiment. For example, different operators may have different operating habits, time management methods, and understanding of experimental conditions. These differences can lead to inconsistencies in experimental conditions and subsequently affect the results. To minimize the impact of these differences on the experimental results, strict experimental operating standards and procedures have been established to ensure the reliability and reproducibility of the experimental results. Additionally, clear instruction is present on the surgical incisions and implantation areas, which facilitates the construction process of the model and reduces variations among different operators, thereby improving the accuracy and reproducibility of the model. Moreover, these clear standards are easily identifiable by skilled surgeons and beginners alike, which helps shorten the surgical time and reduces the risk of surgical failure due to excessively long operation duration.

### Limits and applications

The proposed model is promising for applications for the following reasons. First, the design of animal models should follow the 3R principles [21,47,48]. In this study, induction of peri-implant inflammation has not been able to replace animal studies by organ-on-a-chips technology or computer simulation; our model has a high success rate of osseointegration that can reduce the numbers of rat; and refined surgical procedures can reduce harm to animals and improve animal welfare. Second, animal models need to better mimic clinical situations. In this study, our implant sites and surgical procedures are highly consistent with implant surgery in humans, and the mechanism of inflammation induction is bacterium, which is similar to PI in humans. Therefore, this model can better mimic human physiological and pathological processes well. Considering the multifaceted nature of PI, it is apparent that the utilization of diverse methods to induce the disease is crucial in addressing various research inquiries, such as the physicochemical properties of titanium or its particle release. In future research, other methods of inducing inflammation need to be further explored. Third, large data can be obtained from animal model without too much time [49]. This model does not need to tooth extraction and therefore saves 8 weeks of healing time. Fourth, the cost of animal access and care, animal availability, social acceptability, tolerance for housing, and convenience of placement should also be considered when design an animal model [50]. Rat has been widely accepted as laboratory animals and has many well-established facilities for housing and transporting, so the model of this study does not have the problems above. In summary, the requirements of researchers are well satisfied by the animal model proposed in this study.

However, it is important to note that while much effort has been made to mimic the clinical situation, PI is a highly complex class of diseases. There are many other influencing factors, including iatrogenic factors, so it is still difficult to completely replicate in rodents. In addition, as an important index to measure the strength of osteointegration, torque data were not collected in this study, and it worth to make more effort to obtain in future studies. More efforts are still required for further development of specific small-animal models.

Trans-soft tissue implants PI possess several unique characteristics. It shares with a main common feature: metal implants penetrate soft tissue and bind to bone below for function. TOI consists of bone-anchored part and soft tissue-integration part; thus, the TOI PI can simultaneously result in soft and hard tissue inflammation and destruction. In addition, the TOIs need to penetrate through the soft tissue barrier to exert functions; bacterial assault around the soft tissue sealing area is the leading cause for the development and progression of TOI PI.

**Fig. 7.**
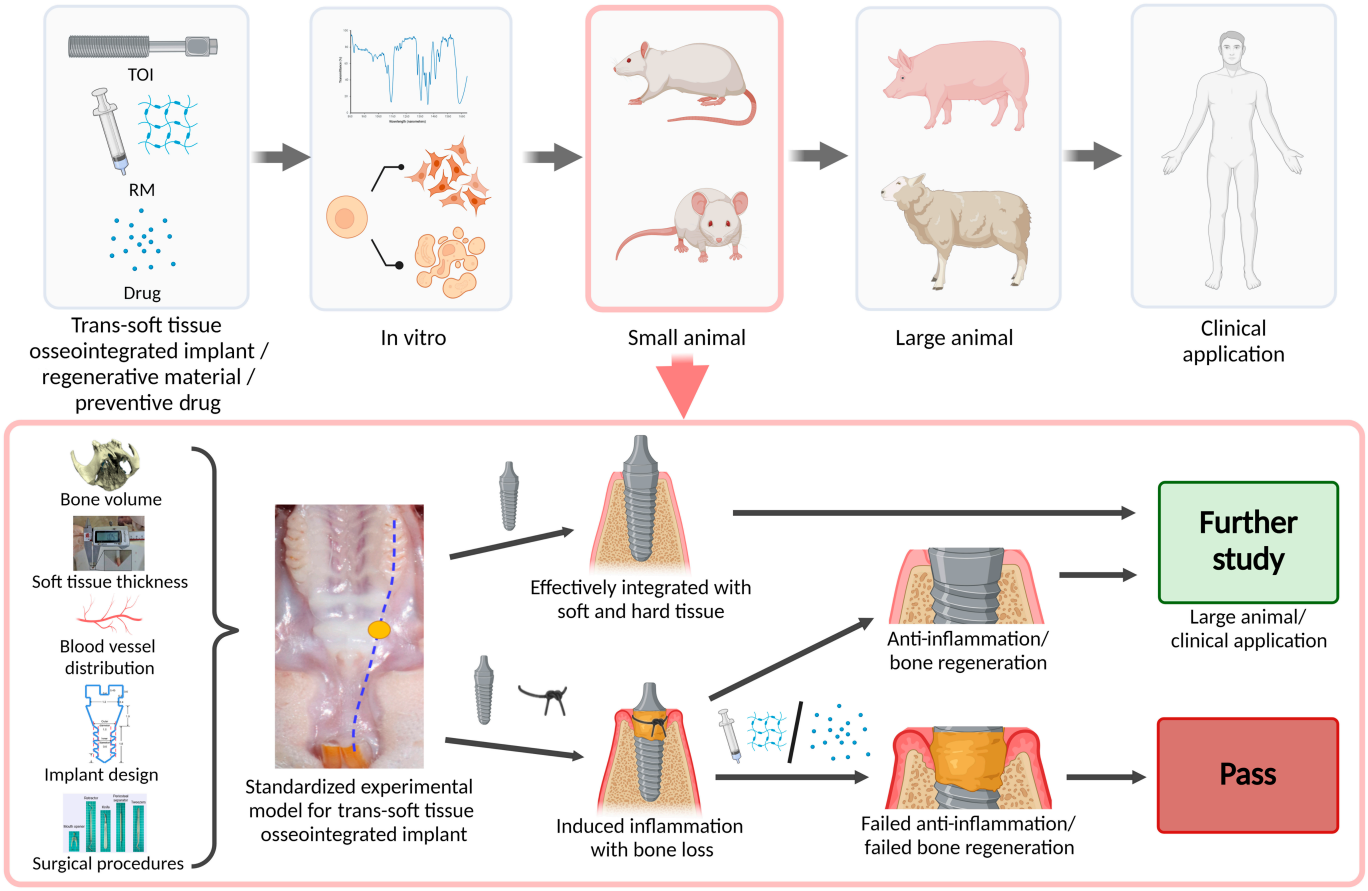
Potential applications of this animal model. This animal model serves as a small-animal model of TOIs to become a crucial bridge between studies in vitro and large animal. It includes rigorous anatomy analysis, scientific implant, and surgical procedures design and can be used to evaluate the osseointegration or anti-inflammation performance of implants with different materials and surface treatment. Besides, it can be used to evaluate the efficacy of nonsolid regenerative materials (RM) or drugs for chronic inflammation and tissue regeneration as a preliminary screening before large-animal and clinical application.

The experimental tools currently available to investigate TOI PI include implants in the forelimb model, implants in periauricular region model, and oral implant model [5,24–26,51,52]. Forelimb model allows force analysis of prosthetic limbs, but amputation of animals is much more harmful than implant in oral. The oral implant animal model has been well accepted as the representative model for the study of TOI. On the one hand, oral implants are one of the most well-known and widely used TOI clinically and possess the typical characteristics such as implant–bone interface and implant–soft tissue interface. On the other hand, a variety of methods (such as ligation [24–26], gingival lavage with bacterial solution [28], LPS injection of peri-implant soft tissue [27], and implant surface biofilm cultivation [23]) have been developed to induce soft tissue infection of oral implant in animal, thus allowing mimicking the PI of TOI. Therefore, this model could provide valuable insights for the study of TOI (Fig. 7).

However, it is important to note that bone tissue in different parts of the body has different origins. The facial bones originate from neural crest tissue [46], while the majority of other bones derive from the mesoderm [47]. Additionally, there are variations in the mechanical loads, blood supply, soft tissue coverage, and microbial environment among different regions bone. Meanwhile, there are some histological differences between skin soft tissue and mucosal soft tissue, and the corresponding major pathogens are also different. Considering the differences between the mucosa–implant interfaces and skin–implant interfaces, whether this model could fully represent the systemic TOI model remains a question that needs to be considered.

Laboratory animals are essential for translational medicine. Before the clinical translation of novel biomaterials or drugs, it is required that animal experimental studies should be conducted First. In addition to its application in TOI-related research, this standardized small-animal model could also be used for preliminary screening of regenerative biomaterials or preventive drugs and treatment of PI before further large-animal studies and clinical trials (Fig. 7). However, due to the limitations in rat bone mass, this model is more suitable for the evaluation of nonsolid regenerative biomaterials. Besides, the healing potential of this model and its potential application in the evaluation of regenerative therapies still need to be further explored.

To conclude, transmucosal osseointegrated implants are widely used in clinical practice due to their excellent restoration function. However, the chronic inflammation associated with these implants can lead to severe complications and adverse outcomes. Through anatomical dissection of the region anterior to rat maxillary molar, ideal implant site was identified, and matching implants and instruments were designed. Sequentially, the model construction procedure was established, resulting in a standardized rat model of PI with a short experimental period, minimal tissue damage, and high success rate. The establishment of standardized small-animal model contributes to the refinement of in vivo insights for transmucosal-osseointegrated-implants-related research and is a vital step for further studies in large animals and clinical applications.

## Ethical Approval

All experiments were performed with prior approval from the Institutional Animal Care and Use Committee of Sun Yat-sen University (approval no. SYSU-IACUC-2022-001137).

## Data Availability

The datasets used during the current study are available from the corresponding author on reasonable request.
