# Supplementary material for: A Standardized Rat Model to Study Peri-implantitis of Transmucosal Osseointegrated Implants
Source: Biomater Res. 2024 Jun 1;28:0021. doi: 10.34133/bmr.0021 (PMC11142924; doi:10.34133/bmr.0021)
Supplement: Supplementary 1 — Figs. S1 to S11 Tables S1 and S2 [file bmr.0021.f1.zip › Supplementary Materials.docx]

**Supplementary Materials**





Figure S1. Extent and methods of soft tissue thickness measurement. The orange quadrangle shows the position and extent of the soft tissue acquired for measurement. The blue dash line is dentition extension line





Figure S2. The graph depicts the reference lines used to measure bone levels at implants. a, coronal aspect of the implant head; *b,* most apical aspect of the implant tip; *c*, most coronal point of bone-implant contact; *d*, distance between *a* and *b*; and *e*, distance between *a* and *c*. The bone height was calculated using the following formula: 3.8 mm (length of the implant)/*d* × *e*; For each implant, buccal, palatal, mesial, and distal bone levels were measured (average of three technical replicates).



Figure S3. The ROI for bone area and squares for counting inflammatory cells. The ROI was defined by a rectangle that the length of its long side is equal to the length of implant thread segment and transgingival segment, and the long side are 500 μm away from the implant surface. The image of soft tissue defined by four red squares (50 × 50 μm) adjacent to the bone surface and implant surface were further analyzed for inflammatory cells.





Figure S4. EDS mapping of implant sandblasting surface. EDS mapping showed that Al_2_O_3_ particles used in sandblasting were embedded on the surface of the implant, and white arrow indicate the Al_2_O_3_ particles remaining on the surface of the implant resulted in the locally high Al and O element contents.





Figure S5. Instruments and drugs required for implant surgery.





Figure S6. Vascular perfusion results in the maxillary region. B, C, D, and E are the pictures respectively observed from various direction showed in A. The vessel are marked in red. There is a large vessel shown on the buccal side of the alveolar ridge in pre-molar region (yellow arrow), and there are vessels on the superficial of palatal cleft (green arrow).





Figure S7. The position of incision and implant region. (A) The position of incision. The starting point of the incision is 2 mm mesial to the gingival entry point of the first molar and slightly palatal to the extension line of the dentition. Yellow zone: the ideal incision position; black dotted line: the maxillary midline; black dotted circle: palatal cleft, the blue dotted line: the alveolar ridge; the yellow dotted line: the dentition extended line; GEP: gingival entry plane; BSP: bone suture plane; red zone: the buccal and palatal vessels. (B) The position of implant region. Green zone: the ideal implant region; Red arrow: bone suture; D1 = 2mm, a certain distance reserved for the subgingival mesial root; D2 = 6mm to avoid bone suture.





Figure S8. The positional relationship between the implant and the bone surface after implantation (palatal, mesial and buccal perspective from A to C).





Figure S9. Immediate post-operative picture of implant. The transgingival segment allows the ligation groove to expose just above the soft tissue.





Figure S10. The entire model establishment process.





Figure S11. Representative histological image of H&E staining from buccopalatal direction.

Table S1. The primers used for quantitative PCR.

| **Gene** | **Sequence** |
| --- | --- |
| GAPDH | Forward 5’-ACTCCACGACGTACTCAGCG-3’ |
|  | Reverse 5’-GGTCGGAGTCAACGGATTTG-3’ |
| IL-1β | Forward 5’-TCTGTGACTCGTGGGATGAT-3’ |
|  | Reverse 5’-CTTCTTTGGGTATTGTTTGG-3’ |
| IL-18 | Forward 5’-CAACCGCAGTAATACGGAGC-3’ |
|  | Reverse 5’-TCTGGTCTGGGATTCGTTGG-3’ |
| IL-6 | Forward 5’-ACAGTGCATCATCGCTGTTC-3’ |
|  | Reverse 5’-CCGGAGAGGAGACTTCACAG-3’ |
| TNF-α | Forward 5’-GGATCTCAAAGACAACCAAC-3’ |
|  | Reverse 5’-ACAGAGCAATGACTCCAAAG-3’ |

Table S2. Instruments and drugs required for implant surgery.

| **Tool name** | **Vendor (catalog number)** | **Purpose** |
| --- | --- | --- |
| Mouth opener | BIOFIVEN (HH04038) | Keep mouth open |
| Retractor | Kangqiao (E3) | Expose the surgical field |
| Tweezers | Kangqiao | Expose the surgical field |
| Knife | Zhuoyouyue (NO. 3) | Cut the mucosa |
| Periosteal separator | Weirong (6#) | Detach the mucoperiosteal flap |
| Surgical light | Boruida | Make a bright field |
| Operating table | Boruida | Fix the rat |
| Adrenaline | Quanyu | Topical Vasoconstrictors (1:10,000 U/ml) |
| Atropine | Quanyu | Inhibit salivary secretion（0.01ml/kg） |
| Cotton ball | Winner | Wipe away blood and saline |
| Periodontal probe | Shiying | Measure distance |
| Ball drill (Φ=0.5mm) | KOMET (F1) | Make a positioning hole |
| Drill (Φ=0.5/0.7/0.85mm) | UNION TOOL | Prepare the implant hole |
| Power system | Shixin (204) | Prepare the implant hole |
| Irrigator | Kangdelai | Cooling |
| Pre-cooled normal saline | Kelun | Cooling |
| Screwdriver | YUPEI | Screw in the implant |
| Micro tweezers | Kewuo | Fix the implant when screw into hole |
